# Survival of AIDS patients in Sao Paulo-Brazil in the pre- and post-HAART eras: a cohort study

**DOI:** 10.1186/s12879-014-0599-8

**Published:** 2014-11-15

**Authors:** Mariza Vono Tancredi, Eliseu Alves Waldman

**Affiliations:** STD/AIDS Referral and Training Center - Department of Health, R. Santa Cruz, 81, São Paulo, CEP 04121-000 Brazil; Department of Epidemiology, School of Public Health, University of São Paulo, Av. Dr. Arnaldo 715 Cerqueira César, São Paulo, CEP 01246 904 SP Brazil

**Keywords:** AIDS, Survival analysis, HAART, Cohort studies, Brazil

## Abstract

**Background:**

Brazil was the first middle-income country to provide free and universal access to AIDS treatment. Understanding the impact of this policy is key to promote ongoing improvement of current intervention strategies. The aim of this study was to compare mortality rates and survival in a cohort of AIDS patients before and after the introduction of antiretrovirals (ARV) and to investigate predictors of survival.

**Methods:**

A retrospective cohort study of AIDS patients aged 13 years or more living in the city of Sao Paulo was conducted. All patients were recruited from an STD/HIV outpatient clinic between 1988 and 2003 and followed up until 2005. We estimated AIDS mortality rates in person-years (py) and carried out a survival analysis using the Kaplan-Meier method. The Cox proportional hazards model was used to assess predictors of survival in AIDS patients.

**Results:**

The study cohort comprised 6,594 patients. The yearly mean mortality rates were 17.6, 23.2, and 7.8 per 1,000 py for the study periods 1988-1993, 1994-1996, and 1997-2003, respectively. Median survival time was 13.4 and 22.3 months for patients entering the study in the first and second study periods and survival time was 108 months or more in 72% of those entering the study during 1997-2003. Factors independently associated with shorter survival included: AIDS diagnosis during the 1994-1996 (HR 2.0) and 1988-1993 (HR 3.2) periods; 50 years of age or more (HR 2.0); exposure category of injection drug users (IDU) (HR 1.5); 8 years of schooling or less (HR 1.4); no schooling (HR 2.1); and CD4+ counts between 350 and 500 cells/mm^3^ (HR 1.2) and less than 350 cells/mm^3^ at AIDS diagnosis (HR 1.3).

**Conclusions:**

The study showed a strong impact following the introduction of HAART in 1996 with decreased AIDS mortality, increased survival rates, and benefits with early introduction of HAART. However, some groups of patients were less likely to benefit from the new drug regimens. Public policies promoting health equity create an enabling environment helping AIDS control programs in developing countries to achieve their goals as effectively as in developed countries.

**Electronic supplementary material:**

The online version of this article (doi:10.1186/s12879-014-0599-8) contains supplementary material, which is available to authorized users.

## Background

The introduction of highly active antiretroviral therapy (HAART) has dramatically changed the course of the HIV/AIDS epidemic. It has decreased AIDS mortality rates and both increased survival and improved quality of life of people living with HIV/AIDS [1]. Understanding the impact of HAART on the survival of AIDS patients and its predictors is key to promote ongoing improvement of current intervention strategies [2].

Brazil was the first middle-income country to provide in 1996 free and universal access to HAART for AIDS treatment [3]. This policy resulted in increased survival of people living with AIDS, from an estimated five months during the period 1982-1989 [4] to 58 months among new cases diagnosed in 1996 [5]. A recent study of new cases diagnosed in South and Southeast Brazil during the period 1998-1999 showed a survival time of at least 108 months in 50% of patients after diagnosis [6].

There is a need to better understanding the impact of the Brazilian policy of universal access to AIDS treatment as many other developing countries have implemented similar guidelines in recent years. However, most studies in Brazil have focused their analyses on the first years following the introduction of HAART [5],[7].

The aim of this study was to compare mortality rates and survival of AIDS patients before and after the introduction of antiretrovirals (ARV) and to investigate predictors of survival in a cohort of AIDS patients living in the city of Sao Paulo who were diagnosed between 1988 and 2003 and followed as of the end of 2005.

## Methods

A retrospective cohort study of people living with AIDS in the city of Sao Paulo, southeastern Brazil, was conducted. Brazil's largest metropolitan area with a population of around 11 million, the city of Sao Paulo has been hit hard by the HIV/AIDS epidemic accounting for about 15% of all cases reported in the country [8].

**Figure 1:**
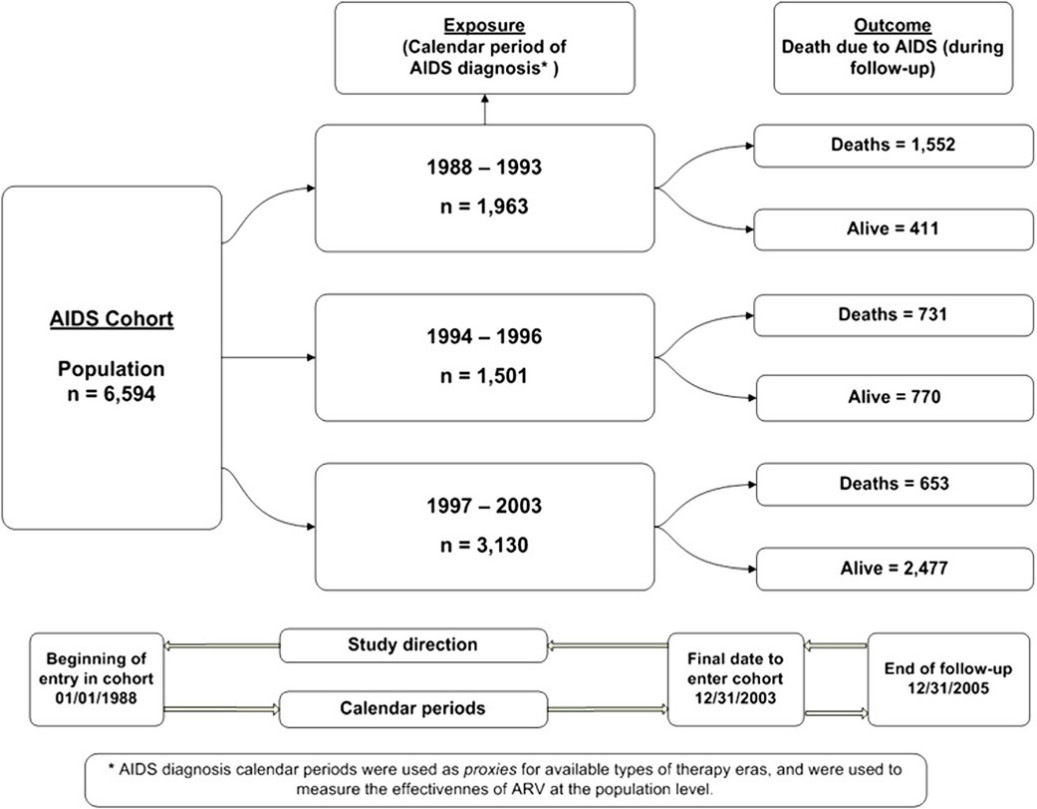
**Study design flow chart for AIDS cohort study.** AIDS Cohort, CRT-DST/AIDS, 1988-2003.

All patients recruited to the study attended the STD/AIDS Referral and Training Center (CRT-DST/AIDS) outpatient clinic. The study cohort comprised AIDS patients aged 13 years old or more living in the city of Sao Paulo with AIDS diagnosis confirmed between January 1^st^, 1988 and December 31, 2003 and followed up until December 31, 2005 (Figure 1). The CRT-DST/AIDS clinic is one of the three specialized centers in Sao Paulo that provide outpatient care to individuals with HIV/AIDS.

The AIDS case definition was any HIV-infected individual with at least one AIDS-defining illness and/or CD4+ counts below 350 cells/mm^3^ regardless of other existing causes of immunodeficiency.

The AIDS case definition for adults changed over the study period following the Brazilian Ministry of Health recommendations: 1) CDC criteria; 2) Rio de Janeiro/Caracas criteria; 3) death exceptional criterion; and 4) adapted CDC criteria. Because there was insufficient information available to apply for all cases the latest AIDS case definition, we chose to use the case definition that was in effect in each period studied [9]. The exposure categories for HIV transmission and treatment regimens were defined according to the Brazilian Ministry of Health criteria [10].

We considered the date of inclusion in the study as the date of confirmation of AIDS diagnosis. Only outpatients enrolled in the CRT-DST/AIDS clinic were included in the study. There were excluded: 1) individuals infected through mother-to-child transmission or blood transfusion; 2) those who died within thirty days after AIDS diagnosis; and 3) those attending a single visit only or followed up for less than 30 days.

The study variables included sociodemographic and behavior characteristics; characteristics related to diagnosis (CD4+ count at AIDS diagnosis) and treatment regimen; and time from AIDS diagnosis to AIDS-related death.

Data was obtained from the following sources of information: CRT-DST/AIDS database (medical and surveillance records); Integrated São Paulo AIDS Database (BIPAIDS) which is a linkage database created from the State Data System Foundation (SEADE) death database and the São Paulo AIDS Program (PEDST-AIDS) Notifiable Diseases Database (SINAN-AIDS) [11]; and the databases of the Brazilian Ministry of Health Laboratory Tests System Control (SISCEL) and Drug Logistics System Control (SICLOM).

The initial cohort included 15,663 individuals enrolled as CRT-DST/AIDS outpatients between January 1^st^, 1988 and December 31, 2003. This database was linked to BIPAIDS, SISCEL and SICLOM databases. Of these, there were excluded 4,398 (28.1%) patients with negative confirmatory HIV tests; 996 (6.3%) who were HIV-infected, but AIDS-free; 634 (4.0%) who were not diagnosed within the study period; 2,814 (18.0%) who attended a single visit only or were followed up for less than 30 days, 147 (0.9%) who were diagnosed with AIDS at death; and 80 (0.5%) who were infected through vertical transmission. The remaining 6,594/15,663 patients (42.1%) were eligible to participate in the study.

### Data analysis

A descriptive analysis of the main patient characteristics was performed in the pre- (1988-1996) and post-HAART periods (1997-2003).

We estimated AIDS mortality rates in person-years (py). The numerator was the number of deaths and the denominator was total person-years at risk of death (in months). The denominator (person-years) was calculated from the date of AIDS diagnosis to the date of AIDS-related death or censoring date.

Data censoring occurred at three different situations: 1) at end of the study period on December 31, 2005 for living patients at follow-up (administrative censoring); 2) at the date of death for those who died from a non-AIDS-related cause; and 3) at the date of the last visit for those lost to follow-up for one year.

We conducted an AIDS survival analysis using the Kaplan-Meier method. Peto and log-rank tests were used to compare time to the event (AIDS-related death) among the groups.

AIDS survival time was estimated from the date of AIDS diagnosis to the date of AIDS-related death or censoring date (in months). Kaplan-Meier curves were generated for the three AIDS diagnosis periods studied: 1988-1993, 1994-1996, and 1997-2003. Combination ARV therapy and HAART became available in the last two study periods, respectively. HAART became available in 1996. These study periods were used as a proxy for different drug regimen exposures and measures of populational effectiveness of these interventions [2],[12]-[14].

The Cox proportional hazards model was used to assess factors associated with time from diagnosis of AIDS to AIDS-related death and to estimate crude and adjusted hazard ratios (HR) with 95% confidence intervals (95% CI). Variables that were statistically significant in the bivariate analysis *(p* < 0.25) and biologically plausible were included in the final Cox proportional hazards model adjusted for treatment regimen. We used the likelihood ratio test to evaluate model fit. Schoenfeld residuals were used to test the proportional risk assumption.

This study was approved by the University of São Paulo School of Public Health research ethics committee (protocol number 1612) and the CRT-DST/AIDS research ethics committee (protocol number 211/06).

## Results

The follow-up of the 6,594 patients with AIDS studied is shown schematically in Figure 1. It shows their distribution according to exposure to different drug regimens and whether or not they died from AIDS-related causes.

A comparison of patient characteristics in the pre- (1988-1996) and post-HAART periods (1997-2003) showed that 52% entered the study in the pre-HAART period.

Compared with pre-HAART, there was in the post-HAART period a lower proportion of injecting drug users (IDU) (13% vs. 28%; *p* < 0.001) and a greater proportion of heterosexuals (45% vs. 28%, *p* < 0.001), which can be explained by an increase in the number of non-IDU women infected, and patients with more than eight years of schooling (61% vs. 46%, *p* < 0.001).

There were no statistically significant differences in CD4+ counts at diagnosis between the first (median 251; first quartile 119 and third quartile 405) and the second period studied (median 220; first quartile 83 and third quartile 363) (p = 0.283). Similarly, there was no significant difference in viral load at AIDS diagnosis between these two periods, ranging from 400 to 100,000 copies/mL in 75% of the patients (p = 0.054). However, it should be noted that only 546/3,464 (15.8%) patients had their viral load measured in the first period.

**Figure 2:**
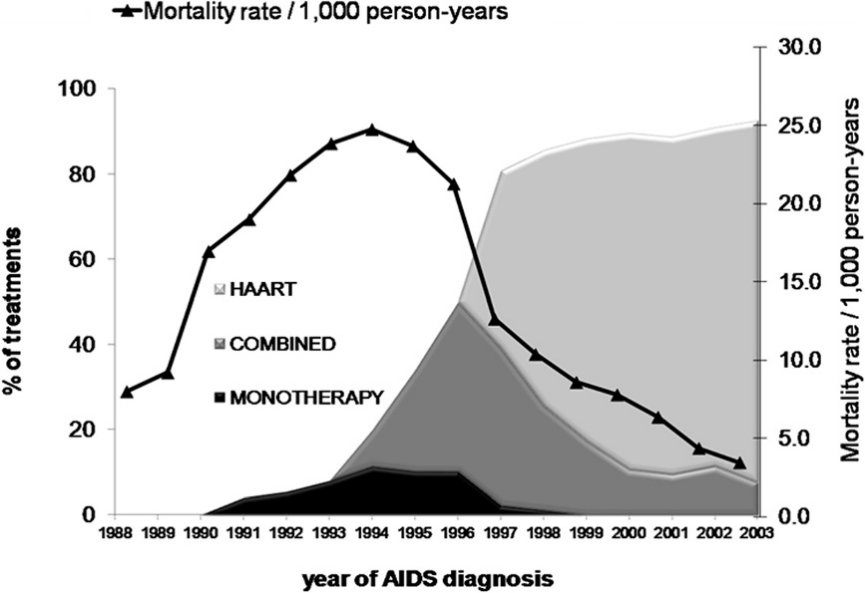
**AIDS mortality rates and proportion of drug regimens prescribed by year of AIDS diagnosis.** AIDS Cohort, CRT-DST/AIDS, 1988-2003.

Of the 6,594 patients studied, 2,936 (44.5%) died during follow-up. From 1988 to 1993, there were 1,552 deaths with a yearly mean mortality rate of 17.6 per 1,000 py. During 1994-1996, there were 731 deaths with a yearly mean mortality rate of 23.2 per 1,000 py, i.e., a 31.8% increase. From 1997 to 2003, there were 653 deaths with a yearly mean mortality rate of 7.8 per 1,000 py, a 66.4% decline in AIDS mortality rate compared to the previous period (Figure 2). Of all patients followed up, 3,698/6,594 (56.1%) were on treatment. Of them, 6.7% received monotherapy, 37.7% combination therapy and 55.6% HAART. Figure 2 shows AIDS mortality rates year by year and the proportion of drug regimen exposure.

The cumulative probability of survival over a maximum follow-up time of 108 months (9 years) was 10.6% and median survival time (MST) was 13.4 months (1.1 years) among patients diagnosed with AIDS from 1988 to 1993. Among those diagnosed from 1994 to 1996, the cumulative probability of survival was 24.4% and MST was 22.3 months (1.8 years). In the last period studied (1997-2003), the cumulative probability of survival over a maximum follow-up time of 108 months was 72.0%. Over 50% patients were alive at the end of the follow-up.

**Figure 3:**
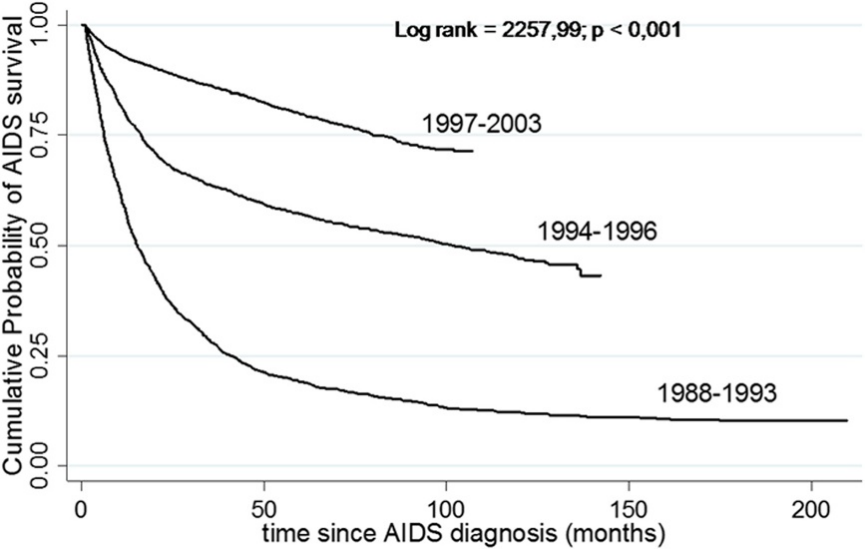
**Cumulative probability of survival in patients diagnosed with AIDS, by study periods.** AIDS-Cohort, CRT-DST/AIDS, 1988-2003.

Figure 3 shows the analysis using Kaplan-Meier curves for the three periods studied (1988-1993, 1994-1996, 1997-2003) which are consistent with the availability of three different drug regimens. Survival increased among those diagnosed with AIDS in the post-HAART period (1997-2003) compared to the other periods studied (log-rank =2,257.9, *p <* 0.001).

**Figure 4:**
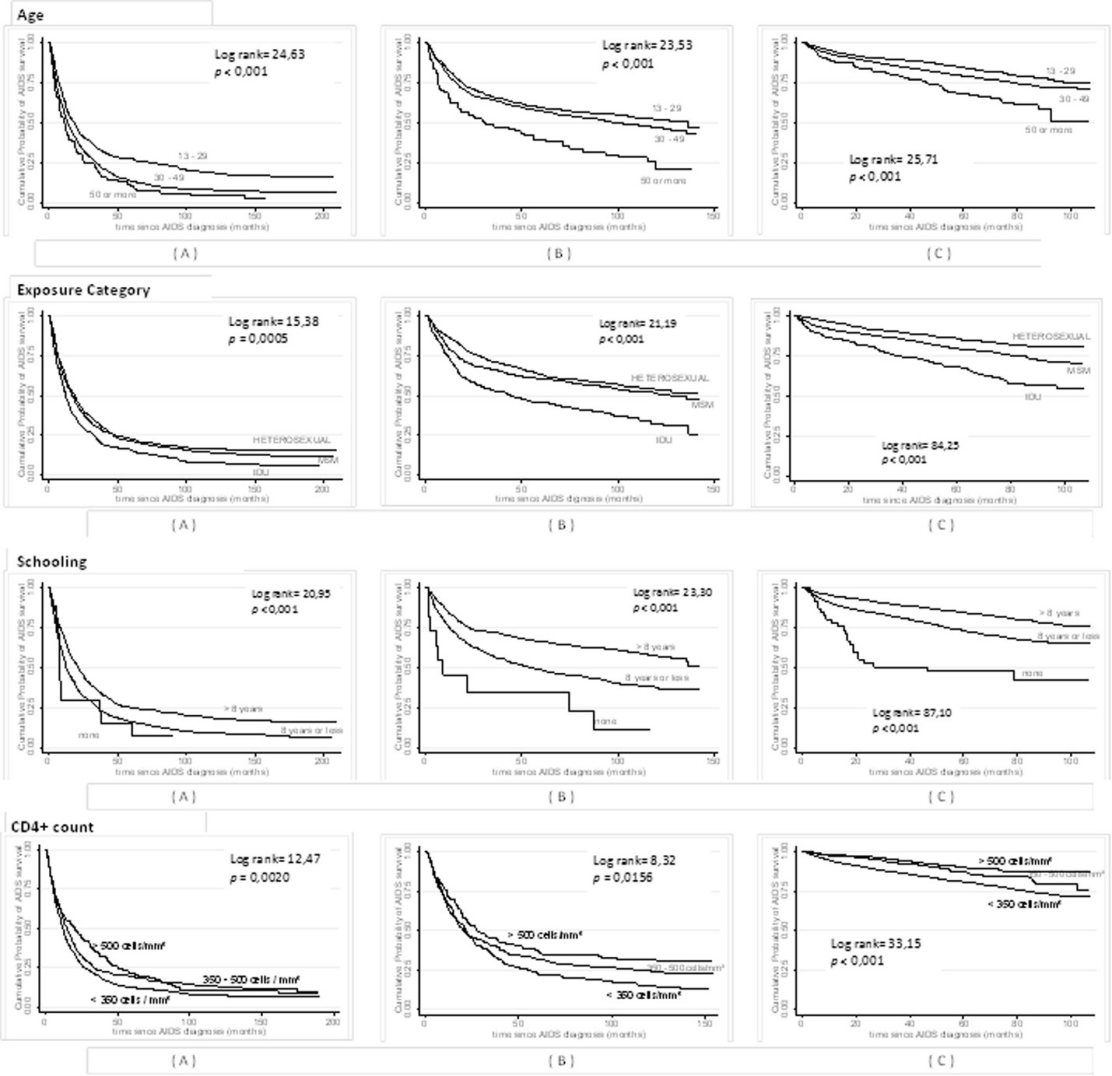
Cumulative probability of survival in patients with AIDS for the three study periods* according to age, exposure category, schooling and CD4+ count at AIDS diagnosis**. AIDS Cohort, CRT-DST/AIDS, 1988-2003. *AIDS diagnosis periods **(A)** = the period from 1988 to 1993, **(B)** =1994-1996; **(C)** =1997-2003. **Log rank tests performed for each variable second periods of diagnosis.

Figure 4 shows increased survival with the use of ARV according to all variables analyzed, with a more pronounced increase among those on HAART.

The Kaplan-Meier survival curve analysis for the post-HAART period showed a decrease in survival with increasing age (log-rank test for trend, *p* < 0.001); greater survival rates in heterosexuals followed by MSM and IDU (log-rank test, *p* < 0.001); and increase in survival rates with increasing schooling (log-rank test for trend *p* < 0.001) and CD4+ count at AIDS diagnosis (log-rank test, *p* < 0.001) (Figure 4).

As for predictors of survival, the bivariate analysis showed the following variables associated with shorter survival (time from AIDS diagnosis to death): AIDS diagnosed from 1994 to 1996 (HR 2.4, 95% CI: 2.2-2.7), and from 1988 to 1993 (HR 6.9, 95% CI: 6.3-7.5); male sex (HR 1.6, 95 % CI: 1.4-1.8); 50 years of age or more (HR 1.4, 95% CI: 1.2-1.6), white skin color (HR 1.6, 95% CI: 1.5-1.8); MSM (HR 1.5, 95% CI: 1.3-1.6) and IDU (HR 2.6, 95% CI: 2.4-2.9); 8 years of schooling or less (HR 2.0, 95% CI: 1.8-2.1); no schooling (HR 2.8, 95% CI: 2.1-3.7); and CD4+ count at AIDS diagnosis between 350 and 500 cells/mm^3^ (HR 1.4, 95% CI: 1.0-1.9) and below 350 cells/mm^3^ (HR 2.5, 95% CI: 1.9-3.2) (Table 1).

**Table 1:** **Bivariate analysis and final Cox proportional hazards model for predictors of survival in AIDS patients**

Characteristics	AIDS	Deaths						
	(n = 6,594)	(n = 2,936)	Crude HR	95% CI	p-value	Adjusted HR	95% CI	p-value
**Study period**					< 0.001			< 0.001
1997-2003	3130	653	1	-		1	-	
1994-1996	1501	731	2.4	2.2-2.7		2.0	1.8-2.2	
1988-1993	1963	1552	6.9	6.3-7.5		3.2	2.8-3.5	
**Gender**					< 0.001			
Female	1526	517	1	-		-	-	
Male	5068	2419	1.6	1.4-1.8		-	-	
**Age** ^**(**^^**&)**^					< 0.001			0.002
13-29	2099	953	1	-		1	-	
30-49	4126	1784	1.0	0.9-1.1		1.4	1.2-1.5	
50 or more	369	199	1.4	1.2-1.6		2.0	1.7--2.3	
**Race/skin color**					< 0.001			
Black/brown	1348	452	1	-		-	-	
White	5157	2456	1.6	1.5-1.8		-	-	
**Exposure category** ^**(**^^**&)**^					< 0.001			< 0.001
Heterosexuals	2310	735	1	-		1	-	
MSM	2698	1215	1.5	1.3-1.6		1.1	1.1-1.2	
IDU	1347	826	2.6	2.4-2.9		1.5	1.3-1.6	
**Schooling** ^**(**^^**&)**^					< 0.001			0.0004
>8 years	3316	1119	1	-		1	-	
8 years or less	2779	1514	2.0	1.8-2.1		1.4	1.3-1.5	
None	78	48	2.8	2.1-3.7		2.1	1.6-2.8	
**CD4+ count** ^**(#)**^					< 0.001			< 0.001
>500 cells/mm^3^	565	56	1	-		1	-	
350-500cells/mm^3^	567	81	1.4	1.0-1.9		1.2	1.1-1.2	
<350 cells/mm^3^	3040	733	2.5	1.9-3.2		1.3	1.2-1.3	

HR: hazard ratio; Crude HR_:_ crude hazard ratio; adjusted HR: adjusted hazard ratio.

^(#)^CD4+ count at AIDS diagnosis; ^(&^^)^ Baseline data.

In the final model, shorter survival was independently associated with the following variables, regardless of other exposures: AIDS diagnosed from 1994 to1996 (HR 2.0, 95% CI: 1.8-2.2) and from 1988 to 1993 (HR 3.2, 95% CI: 2.8-3.5); 30-49 years of age (HR 1.4, 95% CI: 1.2-1.5); 50 years of age or more (HR 2.0, 95% CI: 1.7-2.3 ); MSM (HR 1.1, 95% CI: 1.1-1.2) and IDU (HR 1.5, 95% CI: 1.3-1.6); 8 years of schooling or less (HR 1.4, 95% CI: 1.3-1.5); no schooling (HR 2.1, 95% CI: 1.6-2.8); and CD4+ count at AIDS diagnosis between 350 and 500 cells/mm^3^ (HR 1.2, 95% CI: 1.1-1.2) and below 350 cells/mm^3^ (HR 1.3, 95% CI: 1.2-1.3) (Table 1).

## Discussion

This is one of the few studies examining survival of AIDS patients in Brazil in the pre- and post-HAART eras, most likely the latest available data [4]-[7],[15]. The study results highlight the profound impact of the introduction of HAART on the cohort of AIDS patients studied with a significant decrease in mortality rates and increase in survival. The study cohort accounted for 11.3% (6,594/58,185) of all AIDS cases reported in the city of Sao Paulo during the period studied [16]. Although the sample studied was not representative, our results corroborate those reported by the STD/AIDS Program in Sao Paulo showing a decline in the mortality rate by 68% in the post-HAART period and a 2.8-fold increase in AIDS prevalence resulting from increased survival, despite a 65% decline in new cases [16].

The AIDS epidemic in Sao Paulo has taken a favorable course similar to that seen in developed countries [17],[18]. It reflects the policies implemented in Brazil since 1996 providing free and universal access to HIV/AIDS treatment [7],[15],[19]. As a result, the quality of life of people living with HIV/AIDS has improved and opportunistic infections and hospitalizations have been significantly reduced [20].

In addition to a sharp decline in AIDS mortality and a significant increase in survival time, a key finding of this study is the fact that not all patients benefited equally from new drug regimens and these benefits varied according to individual characteristics of AIDS patients. These differences, however, tended to be less remarkable after the introduction of HAART. The study found that shorter survival time was associated with being diagnosed with AIDS in the pre-HAART period (as discussed before), more advanced age, exposure category of IDU, low schooling and lower CD4+ counts at AIDS diagnosis (a proxy measure for late diagnosis).

The inverse association between age and survival time found in this study is consistent with other studies that reported lower effectiveness of ARV in individuals over 50. These studies have attributed it to higher proportion of late diagnosis in this age group [21],[22], higher prevalence of comorbidity, and decreased immune response with age [18]. This finding is particularly important because recent data from southern Brazil have indicated an increase in new cases among individuals over 50 [23].

Another important finding is that shorter survival time was independently associated with the exposure category of IDU, which is consistent with the literature. A possible explanation may be a higher prevalence of co-infection of HIV and hepatitis B and C in this group [17],[18],[24]. The authors of a study conducted in Sao Paulo argued that it might be due to lower access to HAART and lower CD4+ counts at AIDS diagnosis [25].

The independent association between low schooling and lower survival of AIDS patients may be explained by the fact that disease perception is affected by level of education; educated individuals are more likely to have an early diagnosis and adhere to treatment [25]. If we take schooling as a proxy measure for socioeconomic status, our results are consistent with higher AIDS mortality in the city districts with greater social inequality [26].

The study results indicated an association between low CD4+ counts at AIDS diagnosis and lower survival, which corroborates the findings of several other studies. CD4+ count is a major biological marker of immune status [27]-[29] and an indicator of late diagnosis and untimely treatment. Currently international consensus recommendations for treatment initiation are based on CD4+ counts, viral loads and clinical data [30],[31].

A recent population-based cohort study investigated 5,160 AIDS patients in four of the five Brazilian regions from 2003 to 2010 and found that 53.4% of patients started treatment with CD4+ counts lower than 200 cells/mm^3^ [32], which is close to that seen in our study. It is suggestive of late diagnosis, reinforcing the need to implement strategies for early diagnosis and treatment. Moreover, descriptive data from this same cohort has shown that the groups of patients who benefit less from the introduction of HAART still comprise a significant portion of AIDS patients in Brazil.

It is important to bear in mind the study limitations while interpreting the results. First, data comparisons were made based on the availability of drug regimens at different times rather than on the actual use of these treatments. However, many authors has supported and used this approach [2],[18],[33]. Survival analysis studies often use last known contact date as the censoring date for patients with unknown death information, which can lead to overestimation of survival if a significant proportion of patients with lower survival was lost to follow-up. Another limitation was the use of different AIDS case definitions over time. The use of more sensitive case definitions allowed to diagnose an increased number of cases at a late period, which may have overestimated survival compared to the number of patients diagnosed in the earlier study periods. Despite these limitations, our results are consistent with the literature and support ongoing improvement of AIDS control strategies.

Although factors affecting HAART effectiveness may vary in different populations and backgrounds, our results were obtained under conditions that are in effect comparable to those found in other low- and middle-income countries. These countries have greatly expanded access to ARV treatment in recent years [34], which stresses the valuable contribution of the present study.

## Conclusions

This study emphasizes the impact of HAART introduction, lowering the mortality rates and increasing survival of AIDS patients. It indicated benefits with early initiation of HAART, and identified groups of patients that benefited less from new drug regimens. This finding should be taken into consideration to improve AIDS control programs. Our results also suggest that public policies promoting health equity create an enabling environment helping AIDS control programs in developing countries to achieve their goals as effectively as in developed countries.

## Authors' contributions

MVT and EAW designed and developed the study. MVT and EAW analyzed the data. MVT prepared the manuscript. MVT and EAW discussed data analysis and interpretation. MVT and EAW reviewed the manuscript and approved the final version.

## Authors’ original submitted files for images

Below are the links to the authors’ original submitted files for images. Authors’ original file for figure 1 Authors’ original file for figure 2 Authors’ original file for figure 3 Authors’ original file for figure 4

## Abbreviations

AIDS: Acquired immunodeficiency syndrome
ARV: Antiretrovirals
CD4+: CD4+ T lymphocytes
CDC: Centers for Disease Control and Prevention
CRT-DST/AIDS: STD/AIDS Referral and Training Center
HAART: Highly active antiretroviral therapy
HIV: Human immunodeficiency virus
HR: Hazard ratio
IDU: Injection drug user
KM: Kaplan-Meier
MSM: Men who have sex with men
MST: Median survival time
SEADE: State Data System Foundation
SICLOM: Drug Logistics System Control
SISCEL: Laboratory Tests System Control
STD: Sexually transmitted diseases
