# Supplementary figures and images for: Survival of AIDS patients in Sao Paulo-Brazil in the pre- and post-HAART eras: a cohort study
Source: BMC Infect Dis. 2014 Nov 15;14:599. doi: 10.1186/s12879-014-0599-8 (PMC4247874; doi:10.1186/s12879-014-0599-8)

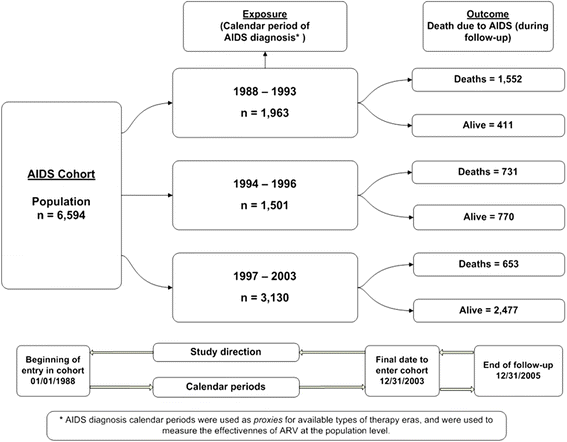

Supplement: Supplementary file 1 — Authors’ original file for figure 1 [file 12879_2014_599_MOESM1_ESM.gif]

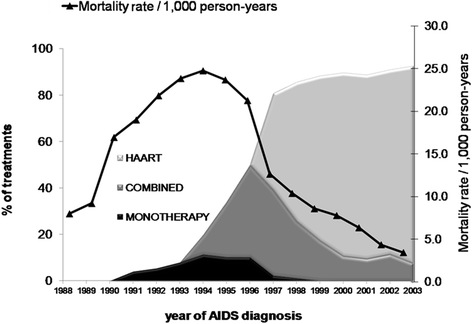

Supplement: Supplementary file 2 — Authors’ original file for figure 2 [file 12879_2014_599_MOESM2_ESM.gif]

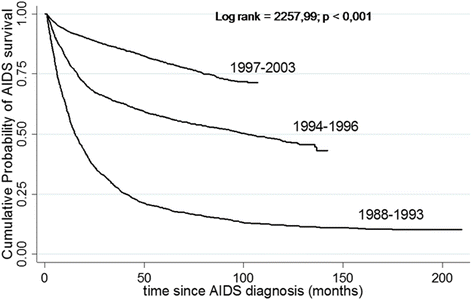

Supplement: Supplementary file 3 — Authors’ original file for figure 3 [file 12879_2014_599_MOESM3_ESM.gif]

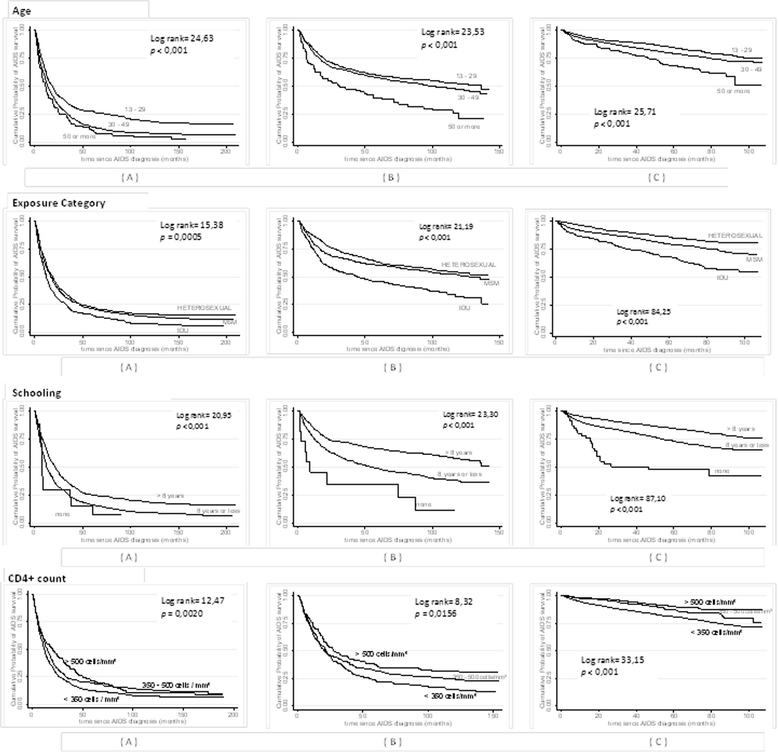

Supplement: Supplementary file 4 — Authors’ original file for figure 4 [file 12879_2014_599_MOESM4_ESM.gif]
